# High STAT4 expression correlates with poor prognosis in acute myeloid leukemia and facilitates disease progression by upregulating VEGFA expression

**DOI:** 10.1515/med-2023-0840

**Published:** 2024-05-09

**Authors:** Aohang Li, Jingxuan Wu

**Affiliations:** Clinical Laboratory Center, Beijing Friendship Hospital, Capital Medical University, Beijing, China; Research Ward, Beijing Friendship Hospital, Capital Medical University, Xicheng District, Beijing, China

**Keywords:** acute myeloid leukemia, poor prognosis, STAT4, VEGFA, apoptosis, angiogenesis

## Abstract

The aim of our study is to explore the mechanism of transcription-4 (STAT4) in acute myeloid leukemia (AML). STAT4 level in AML bone marrow samples/cells was analyzed using bioinformatics and quantitative real-time PCR. The correlation between high STAT4 expression and the prognosis of AML patients was analyzed. The viability, apoptosis, and angiogenesis of AML cells were detected. The levels of STAT4, vascular endothelial growth factor A (VEGFA), and apoptosis-related proteins (Bcl-2 and Bax) in transfected AML cells were examined. STAT4 level was upregulated in AML. STAT4 silencing decreased the viability and angiogenesis, yet increased the apoptosis of AML cells, while overexpressed STAT4 did conversely. VEGFA silencing counteracted the impacts of overexpressed STAT4 upon promoting viability and angiogenesis as well as repressing the apoptosis of AML cells. High STAT4 expression was correlated with poor prognosis of AML patients and facilitated disease progression via upregulating VEGFA expression.

## 1 Introduction

Acute myeloid leukemia (AML) is a hematological malignant tumor characterized by uncontrolled proliferation of hematopoietic stem cells, which can lead to serious consequences such as hematopoietic insufficiency [1,2]. AML can occur at all ages, especially in the elderly, which is coupled with poor prognosis [3]. To date, the pathogenesis of AML is unknown, and previous studies confirm that the pathogenesis of AML may be related to factors, such as chemical poisons, ionizing radiation, and genetics [4,5,6]. Treatment methods for AML include chemotherapy, supportive care, and hematopoietic stem cell transplantation [7,8]. Combined chemotherapy can achieve sustained complete remission in some patients, but the recurrence rate is high, and the toxic and side effects of combined chemotherapy are greater [9,10]. At present, scientists have tried to develop a variety of small molecule targeted therapeutics; however, more in-depth studies should be performed to achieve better results [11]. Therefore, it is important to find new targets for the treatment of AML.

In recent years, with the development of sequencing technology and the application of targeted drugs, the pathogenesis of AML has been further studied [12]. Signal transducer and activator of transcription (STAT) is a kind of DNA-binding protein that plays an important role in immune response, growth, differentiation, proliferation, apoptosis, and tumorigenesis of cells [13,14,15]. Several scholars have shown that STAT5 and STAT3 promote tumorigenesis in myeloproliferative disorders and chronic myelogenous leukemia [16]. Dihydromyricetin enhances the differentiation of AML cells, which is dependent on the activation of the p38–STAT1 signaling pathway [17]. Furthermore, inhibition of the STAT/Ten-eleven translocation 1 axis can be used as a tantalizing therapeutic target in AML by regulating the Janus Associated Kinase (JAK)–STAT signaling [18,19]. STAT4, an important member of the STAT family, has also been found to be closely related to the development of some tumors and autoimmune diseases [20,21]. Zhao et al. found that STAT4 is a key transcription factor for ovarian cancer metastasis, activated STAT4 is overexpressed in epithelial cells of ovarian cancer, and STAT4 overexpression is associated with adverse outcomes of ovarian cancer patients [21]. Notably, it has been found that long non-coding RNAs, which are aberrantly expressed in AML tissues relative to that in normal tissues, can bind to transcription factor STAT4 in a higher ratio [22]. Nevertheless, the detailed mechanism for the involvement of STAT4 in AML awaits to be addressed.

STAT4 assigns a crucial role as a protein of JAK–STAT signaling, which promotes the angiogenesis of glioblastoma [23]. However, it was found that the JAK/STAT pathway is involved in the stimulation of vascular endothelial growth factor A (VEGFA) gene expression. Interestingly, VEGFA-mediated non-cell-intrinsic mechanism has been reported to be involved in murine AML [24]. In previous studies, VEGFA promoter possesses two potent binding positions (BS1 and BS2) for STAT3 [25]. VEGFA is also regulated as a downstream molecule of STAT1 and STAT5 [26,27,28,29]. Therefore, VEGFA was singled out as the downstream factor of STAT4 to dig out the effect of STAT4 on AML cells in this study.

Our current research aims to unveil the impacts of STAT4 upon AML cells by regulating VEGFA expression, so as to seek a potential therapeutic target for AML.

## 2 Methods and materials

### 2.1 Bioinformatics analysis

STAT4 expression in AML bone marrow samples and normal bone marrow samples was analyzed by GEPIA2 (http://gepia.cancer-pku.cn/) and based on the TCGA (https://portal.gdc.cancer.gov/) database.

### 2.2 Cell culture

Human AML cell lines including HL60 (CL-0110), KG1 (CL-0132), Kasumi-1 (CL-0556), and NB4 (CL-0676) were purchased from Procell (Wuhan, China), and normal bone marrow cell line HS-5 (CRL-11882) was ordered from American Type Culture Collection (ATCC; Maryland, USA). In addition, HL60 cells were maintained in HL60 cell-specific culture medium (CM-0110; Procell). Kasumi-1 cells were cultured in Kasumi-1 cell-specific culture medium (CM-0556; Procell). NB4 cells were seeded within NB4 cell-specific culture medium (CM-0676; Procell). KG1 and HS-5 cell lines were maintained in Dulbecco’s Modified Eagle’s Medium (30-2002; ATCC) containing 10% fetal bovine serum (FBS; C0227; Beyotime, Shanghai, China) and 1% penicillin–streptomycin solution (C0222; Beyotime). Moreover, human umbilical vein endothelial cells (HUVECs, CL-0122; Procell) were obtained for tube formation assay and cultivated in HUVEC-specific culture medium (CM-0122; Procell). All cell lines were cultured in the incubator (BC-J250; Boxun, Shanghai, China) at 37℃ with 5% CO_2_, and the culture medium was changed every 2 days.

### 2.3 Cells transfection

HL60 and NB4 cells (1 × 10^6^ cells/well) were divided into two parts and grown in 6-well plates until reaching 80% confluence. In the first part, the plasmid overexpressing STAT4 which was cloned into pcDNA3.1 vector (VT1001; Youbio, Changsha, China), empty vector as the negative control (NC) for STAT4 overexpression plasmid, small-interfering RNA (siRNA) targeting STAT4 (siSTAT4; target sequence: 5′-TTGACAATTGCTTCATTTTAACC-3′), and negative control of siSTAT4 (siNC; 5′-UUCUCCGAACGUGUCACGUTT-3′) were synthesized by Genepharma (Shanghai, China) and transfected into HL60 and NB4 cells, respectively. In the second part, NC and siRNA targeting VEGFA (siVEGFA; target sequence: 5′-TGCTGGAATTTGATATTCATTGA-3′) or siNC, or STAT4 overexpression plasmid and siVEGFA or siNC were co-transfected into HL60 and NB4 cells, respectively. The transfection was performed with the Lipofectamine™ 3000 Transfection Reagent (L3000150; Thermo Fisher, Massachusetts, USA), and all cells were transfected for 48 h.

### 2.4 3-(4,5-Dimethylthiazol-2-yl)-2,5-diphenyltetrazolium bromide assay (MTT) assay

Normal bone marrow cell line HS-5, AML cell lines (HL60, KG1, Kasumi-1, and NB4), as well as transfected HL60 and NB4 cells were collected and cell concentration was adjusted. Then, cells were cultured in 96-well plates for 24 or 48 h according to the instructions of MTT assay kit (C0009S; Beyotime). Next, 10 μL of MTT solution was added to the sample wells for further 4 h of incubation in the incubator. Thereafter, the cells were treated with 100 μL of Formazan solution and cultured in an incubator until Formazan was completely dissolved. Finally, the optical density values of sample wells were determined at a wavelength of 570 nm using a microplate reader (PLUS 384; Molecular Devices, California, USA).

### 2.5 Flow cytometry assay

After the transfected HL60 and NB4 cells were cultured for 24 h, all cells from each group were collected into centrifuge tubes to prepare cell suspension with appropriate amount of culture medium and adjust the cell concentration. Following this, 5 × 10^5^ cells were aspirated from each group of transfected cells and washed with phosphate-buffered solution (PBS) (P301981; Aladdin, Shanghai, China). After the cells were centrifuged (1,000 × *g*) with a centrifuge (75002401; Thermo Fisher) for 5 min, the supernatant was discarded and 195 μL of AnnexinV-FITC conjugate solution (C1062S; Beyotime) was added to resuspend the cells. Subsequently, appropriate amounts of propidium iodide and Annexin V-FITC were supplemented to the sample wells, respectively, and the cells were incubated in the dark at room temperature for 20 min. After the completion of incubation, the apoptotic rate of the cells was detected using a flow cytometer (NovoCyte; Agilent, California, USA).

### 2.6 Tube formation assay

Before the tube formation assay, the transfected HL60/NB4 cells and HUVECs were maintained in their corresponding specific culture media and cultured for 8 h to adjust the cell concentration. HUVECs were co-cultured with transfected HL60/NB4 cells in 24-well plates at a density of 2 × 10^4^ cell/well and were placed onto the layer of thawed Matrigel (356234; Molecular Devices) within the medium. Afterwards, HUVECs and HL60/NB4 cells alone or in combination were cultured in an incubator at 37℃ for 24 h to form tubes. Finally, capillary-like structure formation was observed and photographed using an inverted optical microscope (magnification ×100; DMi8; Leica, Weztlar, Germany).

### 2.7 Quantitative real-time PCR (qPCR)

AML cells and normal cells were collected into a centrifuge tube. Then, appropriate amount of total RNA extractor TRIzol (B511311; Sangon Biotech, Shanghai, China) was added into the centrifuge tube to extract total RNA. Next, RNA purity was determined using NanoDrop™ Lite Spectrophotometer (ND-LITE-PR; Thermo Fisher). Reverse transcription of RNA into cDNA was performed using a reverse transcription kit (B639277; Sangon Biotech) according to the product instructions, and all procedures were implemented on ice. The reactions were performed on a qPCR system (11732-927; Applied Biosystems, Foster City, CA, USA) with a Hotstart HiTaq One-Step RT-PCR Mix (B110026; Sangon Biotech). All primer sequences in this experiment were provided in Table 1, the results were analyzed using the 2^−ΔΔct^ method, and glyceraldehyde-3-phosphate dehydrogenase (GAPDH) was adopted as a reference gene.

**Table 1:** All primers in RT-PCR experiments in this study

ID	Forward sequence (5′–3′)	Reverse sequence (5′–3′)
STAT4	TGTTGGCCCAATGGATTGAAA	GGAAACACGACCTAACTGTTCAT
Bcl-2	GGTGGGGTCATGTGTGTGG	CGGTTCAGGTACTCAGTCATCC
Bax	CCCGAGAGGTCTTTTTCCGAG	CCAGCCCATGATGGTTCTGAT
VEGFA	AGGGCAGAATCATCACGAAGT	AGGGTCTCGATTGGATGGCA
GAPDH	GCAAGTTCAACGGCACAG	GCCAGTAGACTCCACGACAT

### 2.8 Western blot

In this experiment, a total protein extraction kit (BB-3101; BestBio, Nanjing, China) was used to extract total protein from transfected HL60 and NB4 cells. Briefly, the transfected cells were collected and centrifuged (1,000 × *g*) for 5 min prior to the removal of the medium. Then, the cells were washed twice with PBS for 3 min each time. After that, the collected cells were placed on ice to prepare protein extraction working solution. Appropriate amount of protein lysate was added to the cells to extract total protein, subsequent to which the concentration was determined by a BCA protein kit (23229; Thermo Fisher). According to the molecular weight of the detected protein, the sodium dodecyl sulfate–polyacrylamide gel electrophoresis gel was prepared with a gel preparation kit (BB-3702; BestBio). Later, 20 μL of protein samples was added into the sample well of gel for the electrophoresis experiment. The protein was transferred onto polyvinylidene fluoride membrane (YA1701; Solarbio) and then blocked with 5% fat-free milk for 2 h. After that, the membrane was rinsed with Tris-HCl buffered saline with Tween-20 (TBST, ST673; Beyotime) and incubated with primary antibodies at room temperature for 1 h. Then, the membrane was further incubated with diluted secondary antibody at 4℃ for 14 h and washed using TBST. Thereafter, an enhanced chemiluminescence kit (32106; Thermo Fisher) was used for visualization after the protein bands were collected. Finally, data on the strips were further analyzed by the automatic chemiluminescence image analysis system (A44240; Thermo Fisher Scientific, USA). All information on antibodies used was listed in Table 2, and GAPDH was applied as the loading control.

**Table 2:** All antibody information and sources in Western blot in this study

ID	Catalog number	Company (country)	Molecular weight (kDa)	Dilution ratio
STAT4	ab68156	Abcam (Cambridge, UK)	86	1/1,000
Bcl-2	ab182858	Abcam (Cambridge, UK)	26	1/2,000
Bax	ab32503	Abcam (Cambridge, UK)	21	1/2,000
VEGFA	ab46154	Abcam (Cambridge, UK)	27	1/10,000
GAPDH	ab8245	Abcam (Cambridge, UK)	36	1/1,000
Rabbit IgG	ab205718	Abcam (Cambridge, UK)		1/5,000
Mouse IgG	ab205719	Abcam (Cambridge, UK)		1/5,000

### 2.9 Statistical analysis

Statistical analysis was performed using SPSS 20.0. Measurement data were expressed as mean ± standard deviation (*n* = 3). The statistical significance of differences was evaluated using repeated measures analysis of variance (ANOVA) for analyzing multiple measurements of the same group at different time points or conditions, and data in multiple groups were compared using one-way ANOVA followed by a Tukey post hoc test. *P* < 0.05 was determined to be statistically significant.

## 3 Results

### 3.1 STAT4 expression was upregulated in AML and highly expressed STAT4 indicated poor prognosis of AML patients

**Figure 1:**
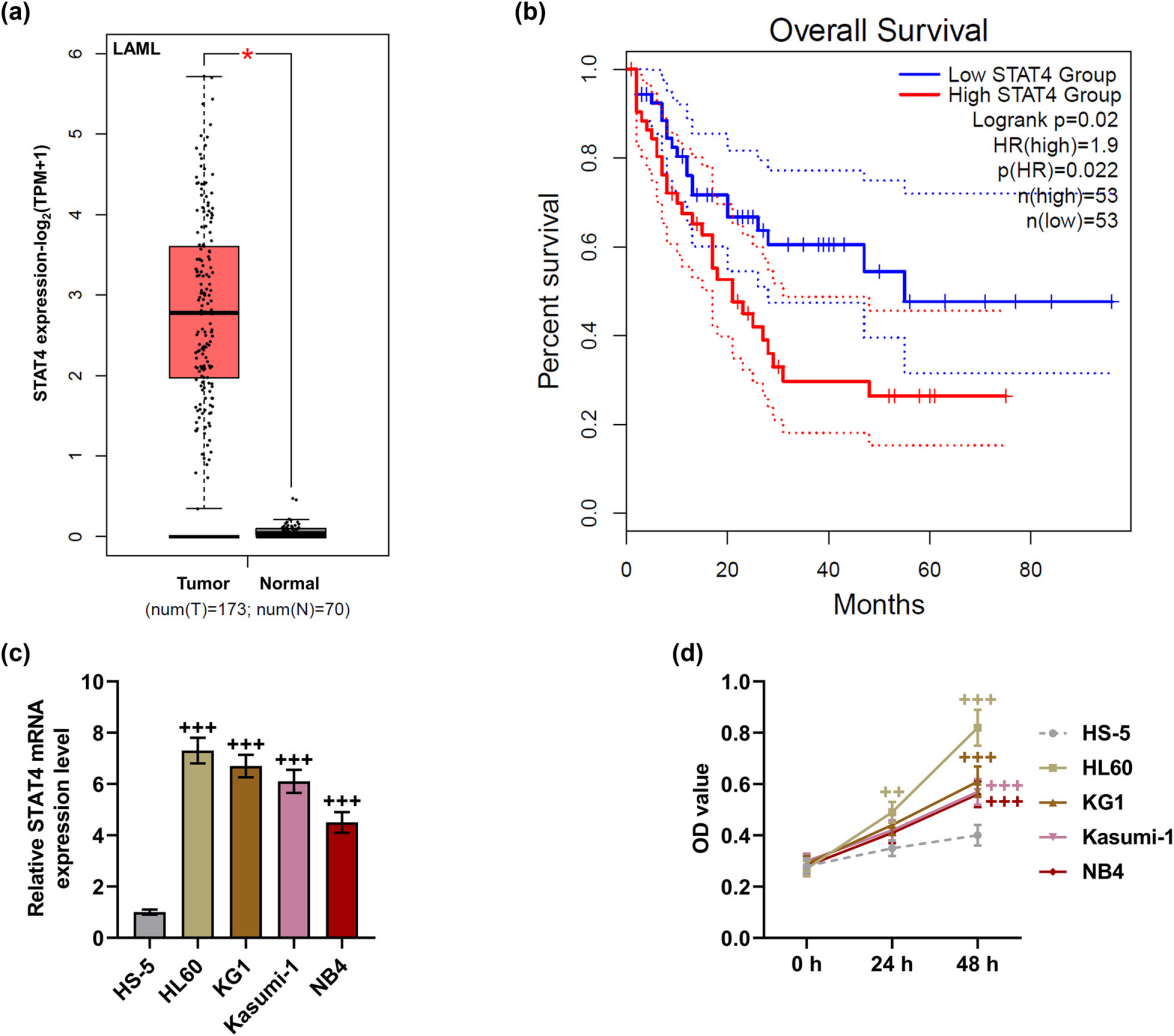
STAT4 expression was upregulated in AML, and highly expressed STAT4 indicated the poor prognosis of AML patients. (a) The expression of STAT4 in AML was analyzed by GEPIA2. (b) The correlation between high STAT4 expression and the prognosis of AML patients was analyzed. (c) The expression of STAT4 in AML cell lines and HS-5 cells was examined by qPCR, and GAPDH was used as a reference gene. (d) The viability of AML cell lines and HS-5 cells was examined by MTT assay. ^+^ *P* < 0.05, ^++^ *P* < 0.01, ^+++^ *P* < 0.001 vs HS-5 (AML: acute myeloid leukemia, GAPDH: glyceraldehyde-3-phosphate dehydrogenase, MTT: 3-(4,5-dimethylthiazol-2-yl)-2,5-diphenyltetrazolium bromide assay, qPCR: quantitative real-time PCR, STAT4: signal transducer and activator of transcription-4).

Bioinformatics analysis showed that STAT4 expression was higher in AML bone marrow tissue samples than in normal bone marrow tissue samples (Figure 1a, *P* < 0.05). Meanwhile, survival analysis mirrored that high expression of STAT4 was positively correlated with poor prognosis of AML patients (Figure 1b). To verify the above predictions, STAT4 level in AML cells was examined. As delineated in Figure 1c, STAT4 level was upregulated in AML cells, when compared with that in the normal cells (*P* < 0.001). In addition, results of MTT showed that the viability of AML cell lines was markedly enhanced in comparison to that of the normal bone marrow cell line HS-5 (Figure 1d, *P* < 0.05).

### 3.2 STAT4 silencing decreased the viability, angiogenesis as well as STAT4 and Bcl-2 levels, yet increased the apoptosis and Bax level in AML cells, while overexpressed STAT4 did conversely

**Figure 2:**
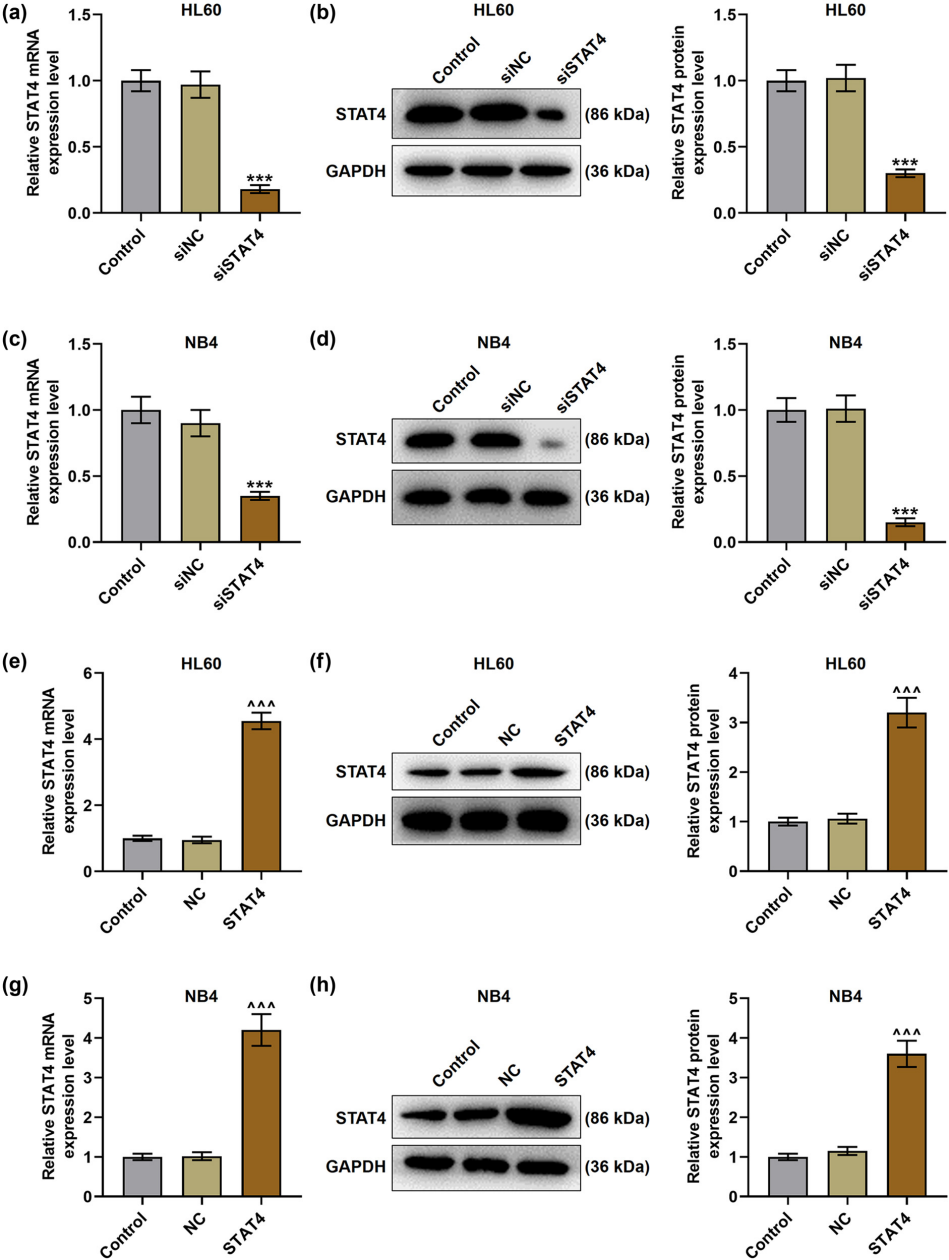
SiSTAT4 decreased STAT4 expression, while overexpressed STAT4 did conversely in transfected HL60 and NB4 cells. (a) The expression of STAT4 in HL60 cells after transfection was examined by qPCR, and GAPDH was used as the loading gene. (b) The expression of STAT4 in HL60 cells after transfection was examined by Western blot, and GAPDH was adopted as the internal control. (c) The expression of STAT4 in NB4 cells after transfection was examined by qPCR, and GAPDH was employed as a loading gene. (d) The expression of STAT4 in NB4 cells after transfection was examined by Western blot, and GAPDH was utilized as the internal control. (e) The expression of STAT4 in HL60 cells after transfection was examined by qPCR, and GAPDH was harnessed as the reference gene. (f) The expression of STAT4 in HL60 cells after transfection was measured by Western blot, and GAPDH was exploited as the internal control. (g) The expression of STAT4 in NB4 cells after transfection was quantified by qPCR, and GAPDH was applied as the reference gene. (h) The expression of STAT4 in NB4 cells after transfection was determined by Western blot, and GAPDH was adopted as the loading control. ^***^ *P* < 0.001 vs siNC; ^^^^^ *P* < 0.001 vs NC (NC: negative control, siSTAT4: siRNA targeting STAT4, siNC: negative control of siSTAT4).

**Figure 3:**
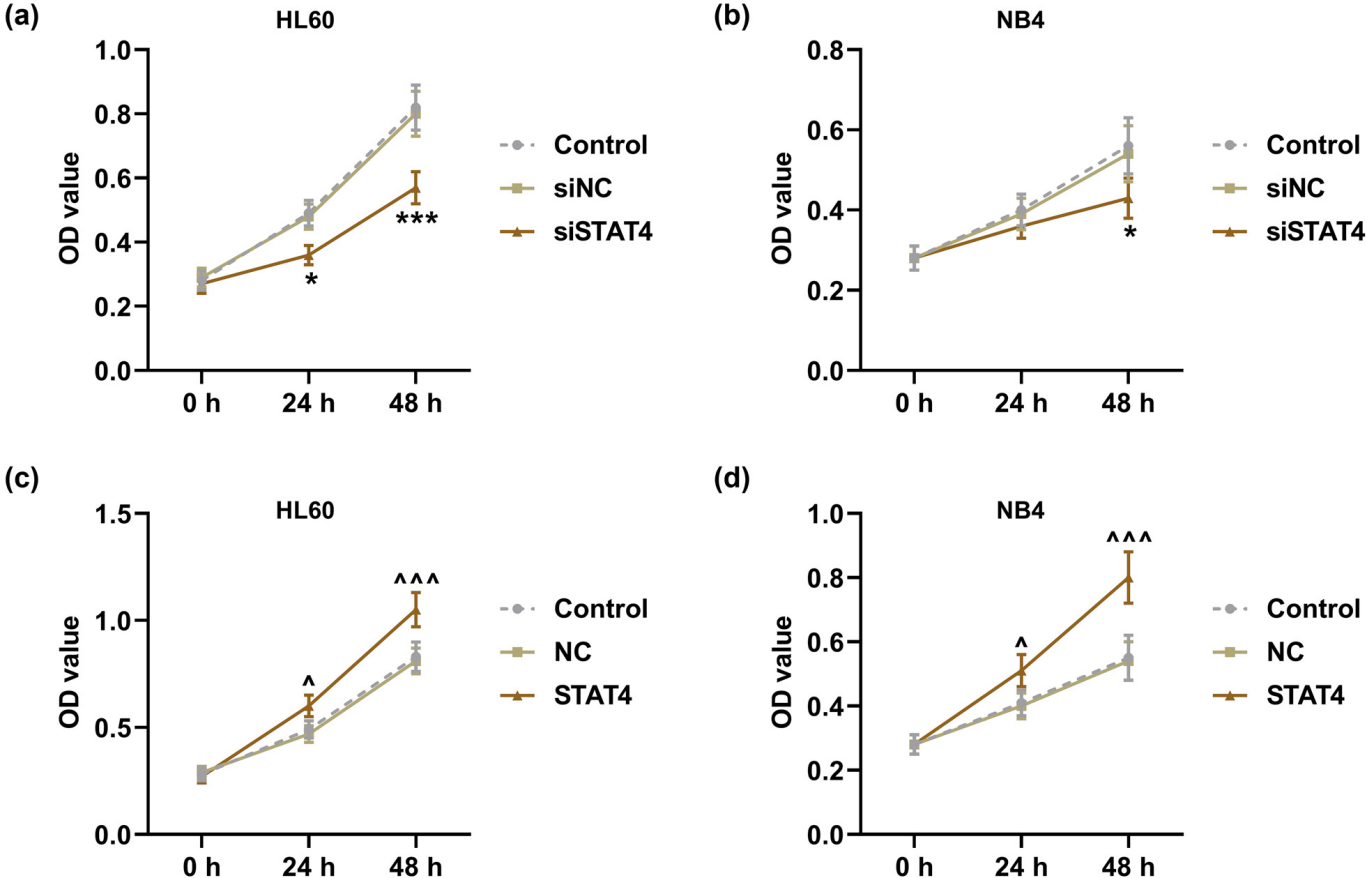
STAT4 silencing decreased the viability, while overexpressed STAT4 did conversely in transfected AML cells. (a–d) The viability of HL60 and NB4 cells after transfection was examined by MTT assay. ^*^ *P* < 0.05, ^***^ *P* < 0.001 vs siNC; ^^^ *P* < 0.05, ^^^^^ *P* < 0.001 vs NC.

**Figure 4:**
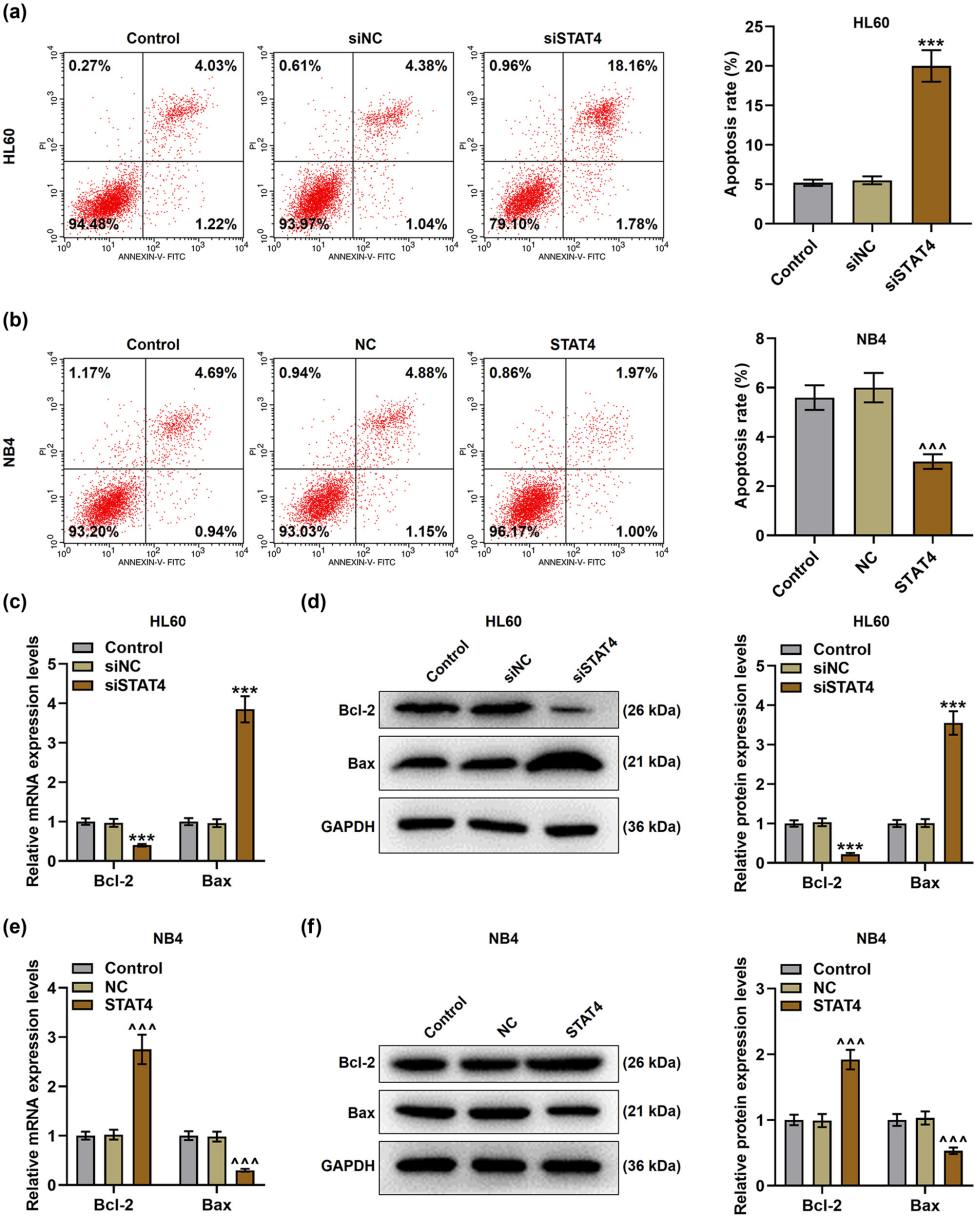
STAT4 silencing decreased Bcl-2 level, yet increased apoptosis and Bax level in AML cells, while overexpressed STAT4 did conversely. (a and b) The apoptosis of HL60 and NB4 cells after transfection was examined by flow cytometry. (c) The expressions of Bcl-2 and Bax in HL60 cells after transfection were quantified by qPCR, and GAPDH was used as the reference gene. (d) The expressions of Bcl-2 and Bax in HL60 cells after transfection were detected by Western blot, and GAPDH was used as the loading control. (e) The expressions of Bcl-2 and Bax in NB4 cells after transfection were measured by qPCR, and GAPDH was used as the reference gene. (f) The expressions of Bcl-2 and Bax in NB4 cells after transfection were examined by Western blot, and GAPDH was used as the internal control. ^***^ *P* < 0.001 vs siNC; ^^^^^ *P* < *P* < 0.001 vs NC.

**Figure 5:**
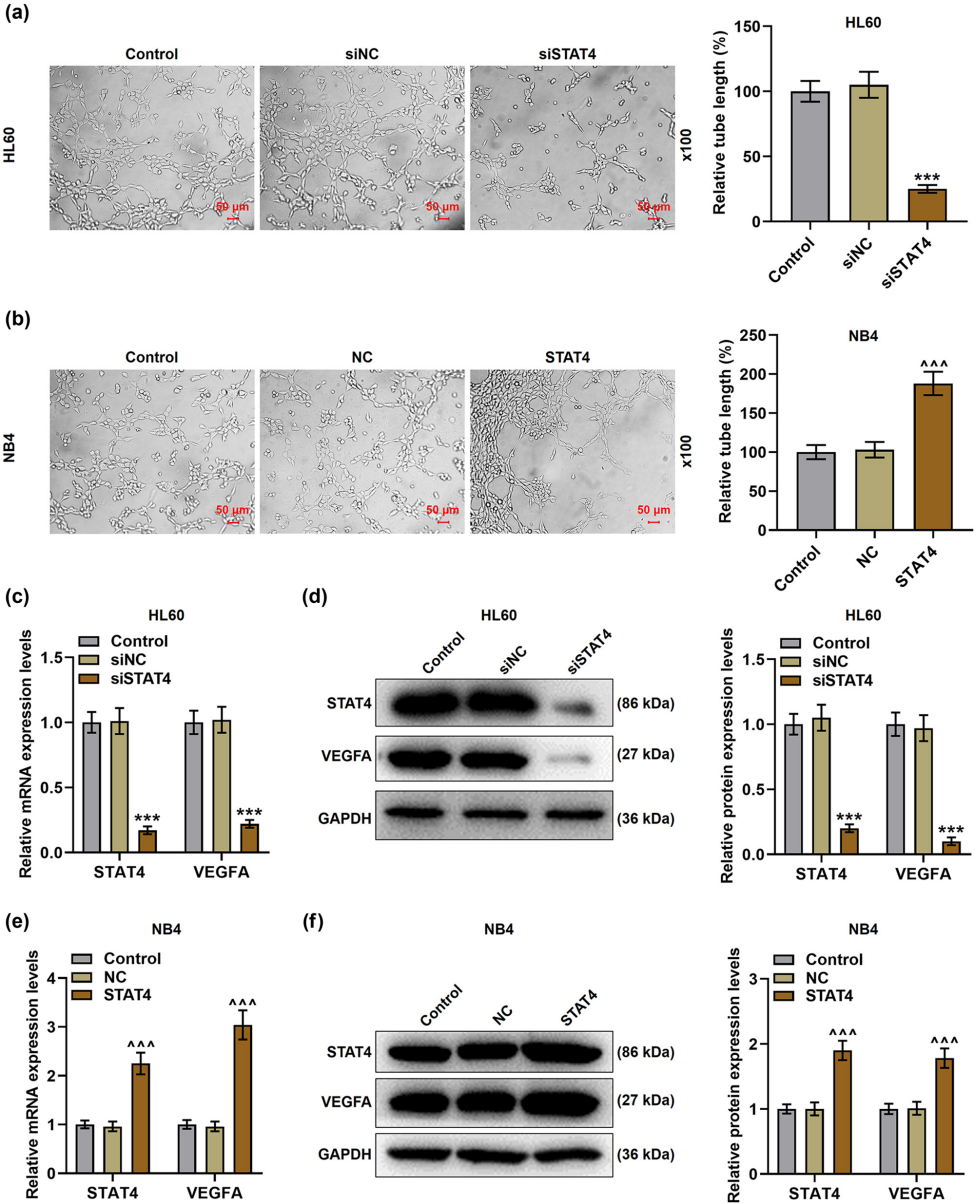
STAT4 silencing decreased the angiogenesis and VEGFA level, while overexpressed STAT4 did conversely. (a and b) The angiogenesis of HL60 and NB4 cells after transfection was examined by tube formation assay under ×100 magnification. (c) The expressions of STAT4 and VEGFA in HL60 cells after transfection were tested by qPCR, and GAPDH was used as the reference gene. (d) The expressions of STAT4 and VEGFA in HL60 cells after transfection were determined by Western blot, and GAPDH was adopted as the internal control. (e) The expressions of STAT4 and VEGFA in NB4 cells after transfection were assayed by qPCR, and GAPDH was utilized as the reference gene. (f) The expressions of STAT4 and VEGFA in NB4 cells after transfection were measured by Western blot, and GAPDH was exploited as the loading control. ^***^ *P* < 0.001 vs siNC; ^^^^^ *P* < 0.001 vs NC (VEGFA: vascular endothelial growth factor A).

The level of STAT4 in transfected HL60 and NB4 cells was detected. In the above two cells, STAT4 expression in the siSTAT4 group was markedly lower than that in the siNC group (Figure 2a–d *P* < 0.001), while STAT4 level in the STAT4 group was markedly higher than that in the NC group (Figure 2e–h, *P* < 0.001). Subsequently, the viability (Figure 3a–d) and apoptosis (Figure 4a and b) of AML cells were detected after transfection. It turned out that siSTAT4 markedly decreased the viability yet enhanced the apoptosis of AML cells in contrast to siNC (*P* < 0.05). On the contrary, overexpressed STAT4 increased the viability but decreased the apoptosis of AML cells relative to NC (*P* < 0.05). In addition, the expressions of apoptosis-related proteins (Bcl-2 and Bax) in transfected AML cells were examined (Figure 4c–f). Strikingly, siSTAT4 clearly reduced the Bcl-2 level yet boosted the Bax expression as compared to siNC (Figure 4c–d, *P* < 0.001). Inversely, overexpressed STAT4 significantly promoted the Bcl-2 level yet inhibited the Bax level in transfected AML cells in contrast to NC (Figure 4e–f, *P* < 0.001). Furthermore, STAT4 silencing decreased the angiogenesis of AML cells (Figure 5a, *P* < 0.001), whereas overexpressed STAT4 significantly promoted the angiogenesis ability of AML cells in contrast with NC (Figure 5b, *P* < 0.001).

### 3.3 VEGFA silencing counteracted the effects of overexpressed STAT4 on promoting the viability, angiogenesis, and Bcl-2/VEGFA level as well as inhibiting the apoptosis and Bax level in AML cells

**Figure 6:**
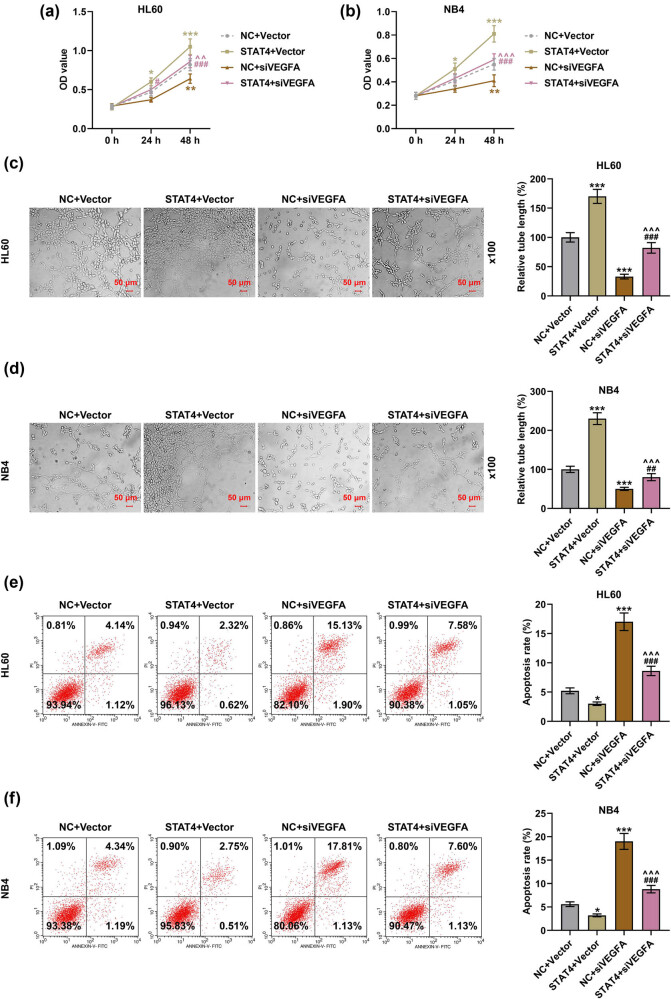
VEGFA silencing counteracted the effects of overexpressed STAT4 on promoting the viability and angiogenesis as well as inhibiting the apoptosis of AML cells. (a and b) The viability of HL60 and NB4 cells after transfection was examined by MTT assay. (c and d) The angiogenesis of HL60 and NB4 cells after transfection was determined by tube formation assay under ×100 magnification. (e and f) The apoptosis of HL60 and NB4 cells after transfection was tested by flow cytometry. ^*^ *P* < 0.05, ^**^ *P* < 0.01^, ***^ *P* < 0.001 vs NC + vector; ^^^ *P* < 0.05, ^^^^ *P* < 0.01, ^^^^^ *P* < 0.001 vs STAT4 + vector; ^#^ *P* < 0.05, ^##^ *P* < 0.01, ^###^ *P* < 0.001 vs NC + siVEGFA (siVEGFA: siRNA targeting VEGFA, vector: negative control for siVEGFA).

**Figure 7:**
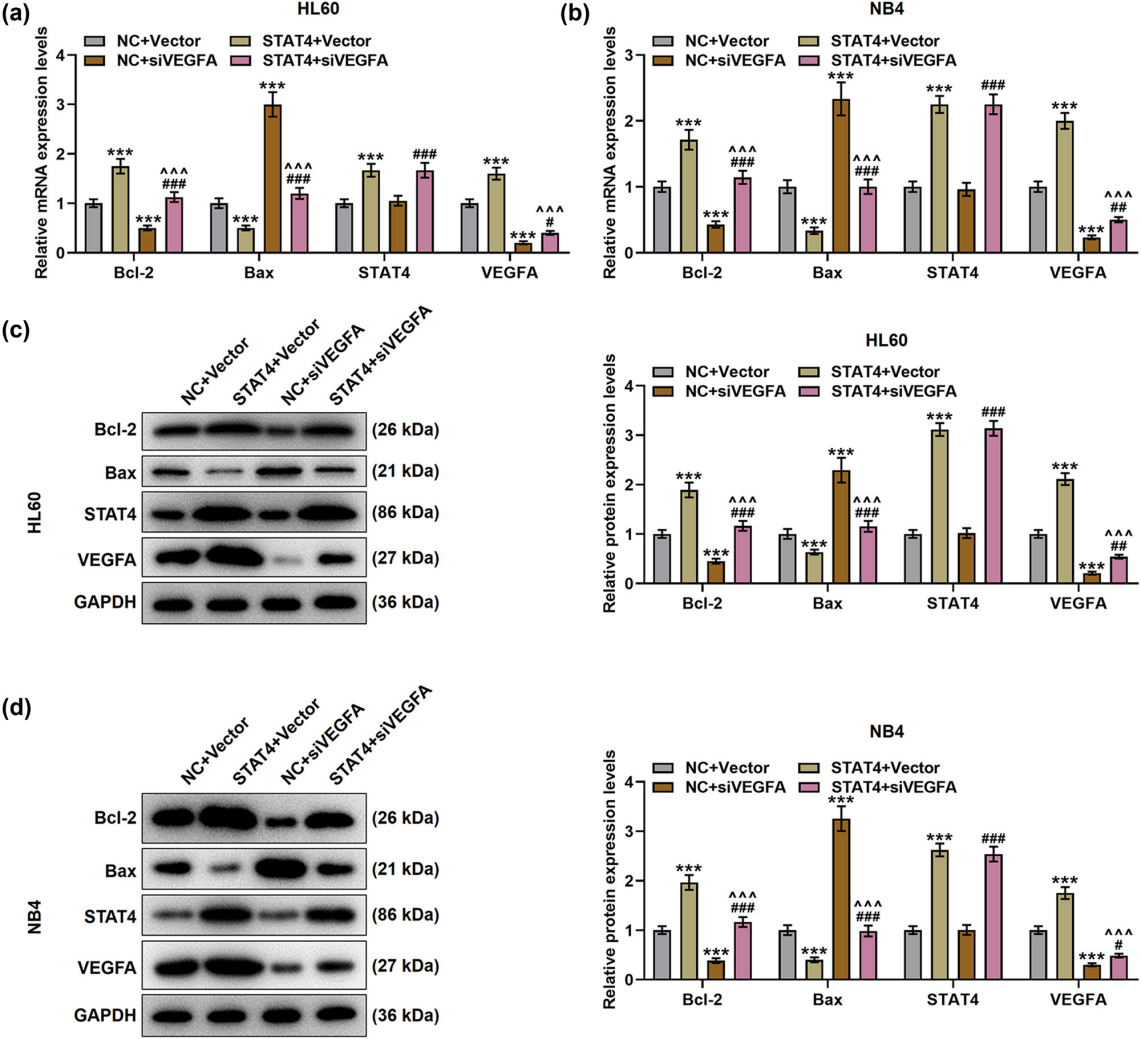
VEGFA silencing counteracted the effects of overexpressed STAT4 on promoting Bcl-2 and VEGFA levels as well as inhibiting Bax level in AML cells. (a and b) The expressions of Bcl-2, Bax, STAT4, and VEGFA in transfected AML cells were examined by qPCR, and GAPDH was used as the reference gene. (c and d) The expressions of Bcl-2, Bax, STAT4, and VEGFA in transfected AML cells were tested by Western blot, and GAPDH was applied as the loading control. ^***^ *P* < 0.001 vs NC + vector; ^^^^^ *P* < 0.001 vs STAT4 + vector; ^#^ *P* < 0.05, ^##^ *P* < 0.01, ^###^ *P* < 0.001 vs NC + siVEGFA.

VEGFA is a key factor in the regulation of angiogenesis [30]. After transfection of siSTAT4, the levels of STAT4 and VEGFA were observed to be downregulated (Figure 5c–d, *P* < 0.001). However, transfection of STAT4 overexpression plasmid increased STAT4 and VEGFA expressions as compared with NC (Figure 5e, f, *P* < 0.001). To further determine the interactions between STAT4 and VEGFA on AML cells, NC and vector/siVEGFA, or STAT4 overexpression plasmid and vector/siVEGFA were co-transfected into HL60 and NB4 cells, respectively. Afterwards, the viability (Figure 6a and b), angiogenesis (Figure 6c and d), and apoptosis (Figure 6e and f) of transfected AML cells were measured. After transfection of STAT4 overexpression plasmid, the viability and angiogenesis of AML cells were boosted; yet, the apoptosis was diminished (*P* < 0.05). On the contrary, VEGFA silencing inhibited the viability and angiogenesis while promoting apoptosis of AML cells in comparison with treatment of NC and vector (*P* < 0.05). Further, siVEGFA reversed the effects of overexpressed STAT4 on the viability, angiogenesis, and apoptosis of AML cells (*P* < 0.01). Moreover, results from qPCR and Western blot suggested that in the presence of overexpressed STAT4, the levels of Bcl-2, STAT4, and VEGFA were raised, but the Bax level was lessened in HL60 and NB4 cells (Figure 7a–d, *P* < 0.001). Inversely, VEGFA silencing repressed Bcl-2 and VEGFA levels, while enhancing Bax level in HL60 and NB4 cells (*P* < 0.001). Meanwhile, siVEGFA counteracted the effects of overexpressed STAT4 on promoting Bcl-2 and VEGFA levels as well as inhibiting Bax level in AML cells (Figure 7a–d, *P* < 0.05).

## 4 Discussion

AML is a genetically heterogeneous clonal disease, whose previous treatment relies on cytotoxic drug-based chemotherapy regimens with difficulties of meeting the clinical treatment needs [31,32]. In recent years, the development of sequencing technology and the application of targeted drugs have provided a new direction for targeted therapy of AML and also brought great progress for the treatment of AML [33,34]. In recent years, great progress has been made in the research and development of inhibitors targeting *fms*-like tyrosine kinase 3 (FLT3) receptor kinase, and FLT3 inhibitors represented by Sorafenib and Midostaurin have been used for the treatment and research of AML [35]. However, single-drug therapy does not significantly improve the survival of AML patients with FLT3-internal tandem duplication mutation [35]. Therefore, it is important to find new therapeutic targets for AML.

Notably, STAT4 is the protein of STAT protein family, and closely related to the development of some tumors and autoimmune diseases, activated STAT4 is overexpressed in epithelial cells of ovarian cancer, and STAT4 overexpression is associated with adverse outcomes of ovarian cancer patients [20,21]. Recent discoveries have highlighted that miR-141-3p represses gastric cancer-induced transition of normal fibroblasts and BMSCs to cancer-associated fibroblasts by targeting STAT4 [36]. Also, high expression of STAT4 remarkably improves the survival rate of patients with breast cancer, especially in aggressive breast cancer subtypes [37]. Moreover, in hepatoma cells, a decrease in STAT4 level indicates a poor prognosis of patients and an enhanced proliferative capacity of cancer cells [38]. In addition, aberrantly expressed long non-coding RNAs in AML have a higher binding ratio to STAT4 [22]. In our experiment, likewise, we found through bioinformatics analysis that STAT4 expression was upregulated in AML, and highly expressed STAT4 indicated the poor prognosis of AML patients. STAT4 silencing decreased the viability and angiogenesis yet increased the apoptosis of AML cells, while overexpressed STAT4 did conversely. The above experimental results illustrated that STAT4 was closely related to the progression of AML. However, the detailed mechanism of STAT4 in AML needs to be further probed.

Increasing evidence has uncovered that Bcl-2 family proteins are closely related to the mitochondrial or intrinsic pathway of apoptosis, where Bcl-2 belongs to a pro-survival protein and Bax protein is a pro-apoptotic protein [39]. In our study, we found that siSTAT4 decreased Bcl-2 level yet increased Bax level in AML cells, while STAT4 upregulation generated a contrary result. It has been underlined that the secretion of tumor angiogenesis-related factors from tumor cells can promote the neovascularization, which is a mechanism for the rapid growth of tumors [40,41,42].

The process of tumor growth has been confirmed to depend on neovascularization which can provide the necessary oxygen and nutrients [43]. Remarkably, VEGFA plays a critical role in the process of angiogenesis [30]. In addition, suppression of VEGFA enhances the invasion and migration while inhibiting the apoptosis of breast cancer cells [44]. Moreover, in colorectal cancer, miR-150-5p inhibits tumor progression by targeting VEGFA; members of the STAT proteins, STAT1, STAT3, and STAT5, have been attested as regulators mediating the expression of VEGFA, involving the mechanisms of angiogenesis as well [25,26,27,28,29]. In our study, we found that the levels of STAT4 and VEGFA were downregulated after transfection of siSTAT4, and VEGFA silencing counteracted the effects of overexpressed STAT4 in AML cells, signifying that STAT4 promoted AML progression via upregulating VEGFA level. These findings provide a novel perspective on investigating the role of STAT4 in the progression of tumors.

Taken together, the role of STAT4 in facilitating AML progression is achieved through upregulating the VEGFA level. Our experimental results, to some extent, open up a new direction for the targeted therapy of AML.
